# Multi‐response optimization of extrusion conditions of grain amaranth flour by response surface methodology

**DOI:** 10.1002/fsn3.1284

**Published:** 2019-11-20

**Authors:** Julian Atukuri, Brian B. Odong, John H. Muyonga

**Affiliations:** ^1^ School of Food Technology Nutrition and Bio‐Engineering Makerere University Kampala Uganda

**Keywords:** complementary feeding, extrusion, gelatinization, grain amaranth, optimization

## Abstract

The study was designed to optimize extrusion processing conditions for production of instant grain amaranth flour for complementary feeding. Multi‐response criteria using response surface methodology and desirability function analysis were employed during the study. The central composite rotatable design (CCRD) was used to determine the level of processing variables and to generate the experimental runs. The process parameters tested included extrusion temperature (110–158°C), screw speed (40–52 Hz), and feed moisture content (11%–16%), while response variable was protein digestibility, sensory acceptability, water absorption index, water solubility index, bulk density, and viscosity. Data obtained from extrusion were analyzed using response surface methodology. Data were fitted to a second‐order polynomial model, and the dependent variables expressed as a function of the independent variables. Analysis of variance (ANOVA) revealed that extrusion parameters had significant linear, quadratic, and interactive effects on the responses. Numerical optimization indicated that the optimum extrusion parameters were extrusion temperature of 150°C, extrusion speed (screw speed) of 50 Hz, and feed moisture content of 14.41%. The responses predicted for optimization resulted in protein digestibility 81.87%, water absorption index 1.92, water solubility index 0.55, bulk density 0.59 gm/L, viscosity 174.56 cP (14.55 RVU), and sensory acceptability score of 6.69, with 71% desirability.

## Graphical abstract

Numerical optimization of grain amaranth extrusion conditions showed that extrusion temperature of 150°C, extruder screw speed of 50 Hz, and feed moisture content of 14.41 % resulted in best product quality parameters. The responses predicted for optimization resulted in protein digestibility 81.87 %, water absorption index 1.92, water solubility index 0.55, bulk density 0.59 gm/L, viscosity 174.56 cP (14.55 RVU), and sensory acceptability score of 6.69, with 71 % desirability. Experimental results confirmed the numerical predictions.

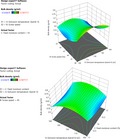

## 1 INTRODUCTION

Malnutrition is problematic during the period of complementary feeding (6–24 months), making this age period crucial in the growth of an infant (Okoth, Ochola, Gikonyo, & Makokha, 2016). Complementary feeding is the delivery of foods or fluids to infants to supplement or replace breast milk (Adepeju, Gbadamosi, Omobuwajo, & Abiodun, 2014; Omueti, Otegbayo, Jaiyeola, & Afolabi, 2009). Most regions facing malnutrition majorly depend on inadequately processed traditional foods mainly comprising cereal gruels from maize, sorghum, and millet (Tou, 2007). Gruels from these cereals form very viscous pastes during cooking and need excessive dilution to suit infant feeding (Ikujenlola & Fashakin, 2005; Kikafunda, Abenakyo, & Lukwago, 2006). However, dilution to reduce thickness results in energy and nutrient thinning which reduces energy and nutrient densities (Amagloh et al., 2013). Furthermore, although cereal crops are widely available, they are inadequate with respect to nutrients sufficiency for infants as cereals mainly consist of carbohydrates and have low levels of protein and minerals (Ijarotimi & Oluwalana, 2013). Malnutrition in developing regions is associated with low nutrient density and bioavailability, time constraint for preparation of food for infants, and inadequate dietary diversity (FANTA‐2010, 2005).

Grain amaranth is desirable because of its nutritional and functional potential, brief growth cycle, and its capability to withstand unfavorable climate and soil conditions (Capriles, Coelho, Guerra‐Matias, & Arêas, 2008a). The entire plant can be used as food (Capriles, Coelho, et al., 2008a). Amaranth seeds contain more protein than other grains and also have high levels of minerals (Kanensi, Ochola, Gikonyo, & Makokha, 2011; Muyonga, Nabakabya, Nakimbugwe, & Masinde, 2008). Amaranth protein is unique because its amino acid balance is close to the optimum for human nutrition (Drzewiecki, 2001). Grain amaranth is a good source of micronutrients especially calcium, iron, potassium, phosphorous, vitamin A, E, and folic acid (Mnkeni, Masika, & Maphaha, 2007; Valcárcel‐Yamani & Lannes, 2012). It has also been shown to contain high levels of phenolics and to exhibit high antioxidant activity (Muyonga, Andabati, & Ssepuuya, 2014), properties associated with prevention of noninfectious chronic diseases such as cancers (Espín, García‐Conesa, & Tomás‐Barberán, 2007). Because of its high content of quality proteins, grain amaranth is highly recommended for complementary feeding (Kanensi et al., 2011). However, grain amaranth is not commonly used for complementary feeding. One of the challenges is the intrinsic taste, nutty flavor, and sandy mouth feel associated with raw grain amaranth (Capriles, Ameida, et al., 2008b; Macharia‐Mutie, Wiel, Moreno‐London, Mwangi, & Brouwer, 2011). Another problem with preparation of complementary foods is the long preparation time, use of more resources in terms of fuel, and high viscosity. Plant‐based complementary foods are often too bulky for the weanling with a tiny stomach to eat the necessary quantities that provide adequate energy and nutrients (Okoth et al., 2016). According to WHO (2003), a good complementary food should produce a gruel that is neither too thick for the infant to consume nor so thin that energy and nutrient density are reduced. Therefore, food processing technologies that improve nutrient density of complementary foods are recommended.

Extrusion technology is a continuous high temperature–short time (HTST) food processing technique that combines mechanical energy with heat energy to gelatinize starch, denature proteins and reorganize food material to form new textured products (Danbaba, Iro, & Mamudu, 2016). Extrusion has high versatility and efficiency, low cost, high output per unit time and leads to very little waste (Nabeshima & Grossmann, 2001). Flours precooked by extrusion can quickly increase their viscosity with low tendency to form clumps in both cold and hot water thus extrusion is recommended for preparation of instant food products (Milán‐Carrillo, Montoya‐Rodríguez, Gutiérrez‐Dorado, Perales‐Sánchez, & Reyes‐Moreno, 2012). Extrusion can also be used to produce low bulk density instant flours which have higher nutrient density than raw flours (Milán‐Carrillo et al., 2012). Extruded products are therefore more suitable for feeding vulnerable persons such as weanlings who are only able to consume limited volumes of food. Changes in extrusion variables such as feed compositions, die configuration, feed rate, and processing temperature among others affect the quality of finished products (Ding, Ainsworth, Tuker, & Marson, 2005). It is therefore crucial to appropriately and effectively optimize production variables during extrusion of products (Danbaba et al., 2016).

Response surface methodology (RSM) and central composite design (CCD) provide an ideal tool for process optimization (Danbaba et al., 2016). Therefore, the aim of the study was to determine the optimal extrusion conditions for production of instant grain amaranth complementary food with high protein content, protein digestibility, sensory acceptability, and low bulk density and viscosity.

## 2 MATERIAL AND METHODS

### 2.1 Processing of extruded grain amaranth flour

Grain amaranth (GA) was purchased from farmers in Uganda through Peak Value Ltd. Grain amaranth was washed, dried, and milled using a commercial mill (30B‐C, Changzhou Erbang Drying Equipment Co. Ltd). The obtained GA flour was then extruded using a corotary and intermeshing twin screw extruder (Double screw inflation food machine DP70‐III, Jinan Eagle Machine Co. Ltd), with specs of 7‐cm screw diameter, 141.7‐cm screw length, and 4‐mm diameter die opening. Extrusion was carried out using different extrusion conditions obtained from design expert^®^ 11.0 (Table 1). Different conditions of extrusion temperature (heating area II), extrusion speed (screw speed), and feed moisture content obtained from the experimental design were used. Preliminary experiments were conducted to determine the range of conditions that gave extrudates with unburnt appearance and no clogging of the product in the barrel. Barrel zone I and zone III temperatures were respectively set 20°C lower and 15°C higher than zone II temperatures. Upon extrusion, the extrudates were collected in polyethylene bags and allowed to cool. The extrudates were then milled into fine flour to produce instant grain amaranth flour. Feed moisture content was measured as the amount of water added to the grain amaranth flour before extrusion, expressed as a percentage of the weight of the flour.

**Table 1:** Independent variables and levels for the response surface design (rotatable central composite design)

Variable	Symbol	Coded variable level				
		−α (−1.4142)	−1	0	1	+α (1.4142)
Extrusion temperature (^o^C)	*X*_1_	101.716	110	130	150	158.284
Extrusion speed (Hz)	*X*_2_	37.9289	40	45	50	52.0711
Feed moisture content (%)	*X*_3_	11.1716	12	14	16	16.8284

### 2.2 Research design for optimization

#### 2.2.1 Experimental design

Response surface methodology (RSM) using the central composite design (CCD) was used to predict the responses. The independent variables were extrusion temperature (heating area II) (*X*_1_), extrusion speed (*X*_2_), and feed moisture content (*X*_3_). Twenty‐six (26) experimental runs were generated using the rotatable central composite design consisting of 8 factorial runs, 12 axial runs, and 6 center runs. The experimental design used to generate the runs was optimized by considering the design with good G‐efficiency and Fraction of Design Space (FDS) score. The independent variables and their levels are shown in Table 1. The response variables (*Y*) considered were protein digestibility, water absorption index, water solubility index, bulk density, viscosity, and sensory acceptability. The dependent variables were individually expressed as a function of the independent variable. A second‐order degree polynomial equation was used for prediction of the response variables (Equation 1).

(1) $$Y=B_{0}+\sum_{i-1}^{k}B_{i}X_{i}+\sum_{i-1}^{k}B_{\mathrm{ii}}X_{i}^{2}+\sum_{\begin{array}{c} i-1\\ i<j \end{array}}^{k}B_{\mathrm{ij}}X_{i}X_{j}+\varepsilon$$

where *Y* is the response function, *B*_0_ the center point of the system, ε is the random error, *B_i_*, *B_ii_*, and *B_ij_* represent the coefficients of the linear, quadratic, and interactive effects, respectively. *X_i_*, *X_i_* ^2^, and *X_i_X_j_* represent the linear, quadratic, and interactive effects of the independent variables (extrusion temperature, extrusion speed, and feed moisture content), respectively.

### 2.3 Optimization of extrusion conditions

The extruded grain amaranth flours were analyzed for protein digestibility, water absorption index (WAI), water solubility index (WSI), bulk density, viscosity, and sensory acceptability. The experiments were randomized to remove bias and reduce systematic errors. The desirability function approach (DFA) was used for optimizing the extruded GA for maximum protein content, protein digestibility and sensory acceptability, desirable WAI and WSI, and minimum bulk density and viscosity.

### 2.4 Physicochemical analyses

#### 2.4.1 Protein digestibility

In vitro protein digestibility (IVPD) was determined according to the method by Mertz et al. (1984). In the first stage, crude protein content was determined as described using Kjeldal method (AOAC, 2005). Flour (0.3 g) was weighed into a Kjeldahl digestion flask and 12 ml of concentrated sulfuric acid added. Selenium was added as a catalyst. The flask was then placed in a digester in a fume hood cupboard and digested for 45 min until a colorless solution was obtained. Distillation was then carried out using 4% boric acid and 20% sodium hydroxide. The distillate was then titrated with 0.6 M HCl until formation of a violet color as the end point. A blank was run under the same conditions. Protein content was measured in triplicate and calculated using the equation below:

(2) $$\text{Protein}\;\text{content}\left(\%\right)=\frac{[\text{titer}\;\text{value}\;\text{of}\;\text{sample}-\text{blank}]\times 0.06\times 14\times 6.25}{1000\times \text{weight}\;\text{of}\;\text{sample}}\times 100$$

The second stage involved digesting the sample using pepsin enzyme. Approximately 0.2 g of sample was weighed and placed in a clean sterile labeled centrifuge tube and 20 ml of buffered pepsin solution added, and the mixture incubated in a water bath at 37°C for 2 hr. The tubes were then centrifuged at 4,500 *g* for 15 min using a FisherScientific centrifuge (ThermoFisher Scientific, 297) resulting into separation of the digestion mixture in two layers. The upper layer was carefully removed using a dropper and discarded. To the remaining solution, 10 ml of 0.1 M potassium dihydrogen phosphate was added, the tube shaken well and centrifuged as before. The mixture was filtered using Whatman No. 3 until all the liquid drained into the test tubes. The filter paper was rolled up and inserted into digestion flasks. The flasks were then dried in the oven at 100°C for 15 min, and later, crude protein content determined following AOAC (2005). Protein digestibility was calculated using the equation below.

(3) $$\text{Protein}\;\text{digestibility}\left(\%\right)=\frac{A-B}{A}$$

where, *A* = % protein in the original sample; *B* = % protein after pepsin digestion.

### 2.5 Water absorption and solubility index

Water absorption index (WAI) and water solubility index (WSI) were determined using the method described by Kaur and Singh (2005). A portion (3 g) of the flour was dissolved in 30 ml of distilled water and heated in a water bath for 15 min at 90°C. The cooked paste was cooled to room temperature, transferred into preweighed centrifuge dishes, and then centrifuged at 1,500 *g* for 20 min. The supernatant was thereafter decanted into a preweighed evaporating dish to determine the solid content and the sediment weighed. The weight of dry solids was obtained by evaporating the supernatant overnight at 105°C. The WAI and WSI were calculated using the equations below.

(4) $$\text{WAI}=\frac{\text{Weight}\;\text{of}\;\text{sediment}}{\text{Weight}\;\text{of}\;\text{flour}\;\text{sample}}$$

(5) $$\text{WSI}\;=\;\frac{\text{Weight}\;\text{of}\;\text{dissolved}\;\text{solids}\;\text{in}\;\text{supernatant}}{\text{Weight}\;\text{of}\;\text{flour}\;\text{sample}}$$

### 2.6 Viscosity

Viscosity was expressed as the final viscosity from the rapid visco‐analysis using a Rapid Visco Analyzer (RVA‐4, Newport Scientific). Grain amaranth flour (5 g) was added to 25 ml of distilled water in a canister and mixed, loaded onto the RVA and run using the extrusion 1 profile. Viscosity was expressed as the final viscosity at a temperature of 49.95°C in centipoise (cP) (1RVU = 12 cP).

### 2.7 Bulk density

Bulk density was determined using a method by Maninder, Kawaljit, and Narpinder (2007). Grain amaranth flour was weighed into a cylinder of known volume in triplicates. The cylinder was gently tapped on a laboratory bench until no further diminution and flour filled to the volume mark. This was done to eliminate air spaces. Weight was measured using a laboratory balance, and the results expressed as weight to volume ratio (Equation 6).

(6) $$\text{Bulk}\;\text{density}\left(g/\text{ml}\right)=\frac{\text{Weight}\;\text{of}\;\text{sample}(g)}{\text{Volume}\;\text{of}\;\text{sample(ml)}}$$

### 2.8 Sensory acceptability

Sensory evaluation was carried out to determine the acceptability of porridges obtained from the extruded grain amaranth flour. Porridges were prepared by adding 33 g of extruded grain amaranth flour to 100 ml of hot water and stirred to uniform and drinkable consistency (2,500–3,000 cP) (Akande, Nakimbugwe, & Mukisa, 2017). A semitrained panel of 25 members consisting 44% males and 56% females (approximately 75% of whom were mothers) was used. Panelists were presented with coded samples and asked to evaluate them according to their preference using a 9‐point Hedonic scale, from 1 (dislike extremely) to 9 (like extremely).

### 2.9 Statistical analysis

Data were analyzed by response surface methodology using design expert statistical software (DX 11.0; Stat‐Ease Inc) to optimize the extrusion conditions of grain amaranth. To estimate the effect of extrusion temperature, extrusion speed and feed moisture content protein digestibility (*Y*_1_), water absorption index (*Y*_2_), water solubility index (*Y*_3_), bulk density (*Y*_4_), viscosity (*Y*_5_), and sensory acceptability (*Y*_6_), the standardized scores were fitted to a quadratic polynomial regression model (Equation 1) (Durgadevi & Nazni, 2012). The statistical parameters used to relate the input variables to the responses were *p*‐value and *R* ^2^. Significance of the models was determined using model analysis and lack of fit.

### 2.10 Validation of RSM results

To validate the mathematical model, the optimal conditions were used for production of extruded flour and the products were analyzed. Predicted were then compared with experimental values. Percentage prediction error was calculated using Equation (7) to validate the model (Scheuer et al., 2016).

(7) $$\text{Predicted}\;\text{error}\left(\%\right)=\frac{\left(\text{experimental}-\text{predicted}\right)}{\text{predicted}}\times 100$$

## 3 RESULTS AND DISCUSSION

### 3.1 Protein digestibility

Protein digestibility is an essential determinant of protein quality. Plant proteins generally have lower digestibility compared with animal proteins due to antinutritional factors such as trypsin inhibitors (Nyakuni et al., 2008; Singh, Gamlath, & Wakeling, 2007). In vitro protein digestibility (IVPD) was determined and the effect of extrusion conditions on protein digestibility is represented in Equation (8), which shows the model in terms of coded levels of the variables. A negative coefficient denotes decrease in the response with increase in the level of the parameter whereas a positive coefficient indicates increase in the response as the level of parameter increases (Filli, Nkama, Jideani, & Abubakar, 2012). Extrusion temperature had a negative linear effect while extrusion speed and feed moisture had positive linear effects on protein digestibility of GA flour.

(8) $$\text{Protein}\;\text{digestibility}=80.29-1.39X_{1}+1.71X_{2}+1.86X_{3}$$

**Figure 1:**
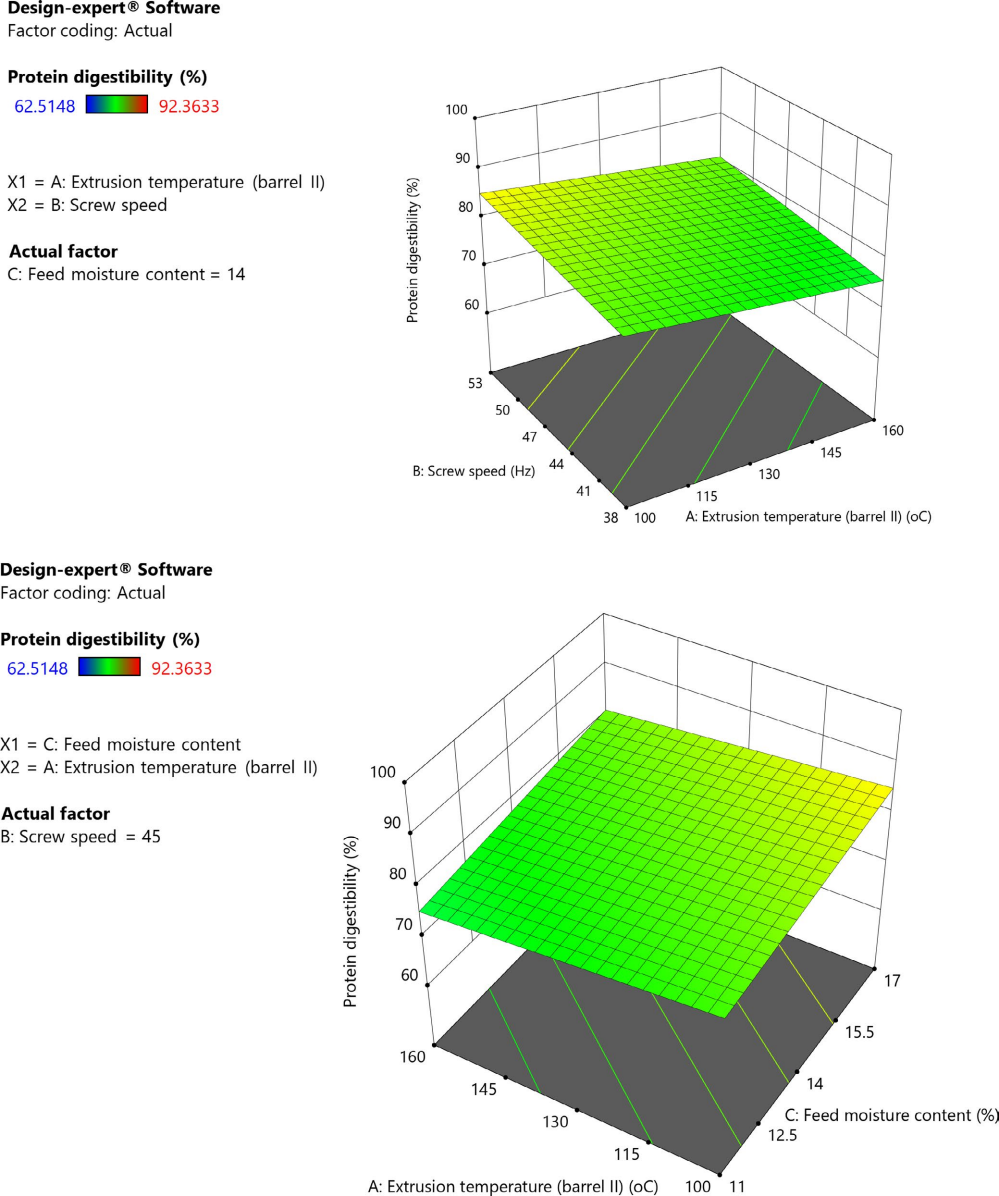
Response surface plot showing effect of extrusion temperature, screw speed, and feed moisture on protein digestibility of grain amaranth flour (Constant moisture (14%), (Constant screw speed, 45 Hz)

Increase in extrusion temperature resulted in decrease in protein digestibility while speed and feed moisture content increased with protein digestibility (Figure 1). The model was insignificant (*p *= .63) and had a nonsignificant lack of fit (*p *= .99). Observed decrease in IVPD with increased extrusion temperature could be due to modifications during extrusion. During extrusion, heating leads to Maillard reactions between the free amino groups of protein and carbonyl groups of reducing sugars (reducing protein digestibility) and also reduces antinutrients which inhibit digestion of proteins by enzymes (Njoki, Silva, & Onyango, 2014; Singh et al., 2007). According to Okpala and Chinyelu (2011), decrease in IVPD results from nonenzymatic browning reactions which cause nonreversible formation of compounds thus a decrease in protein available for digestion. Maillard reactions particularly occur at high temperatures (Peluola‐Adeyemi, Idowu, Sanni, & Bodunde, 2014), and this could explain the decrease in protein digestibility with increase in extrusion temperature. Similar effect of temperature on IVPD was reported by Akande et al. (2017). Screw speed and feed moisture increased protein digestibility because higher shear enhances denaturation of proteins more easily, which facilitates enzyme hydrolysis (Singh et al., 2007). Their disruption aids protein unfolding and thus digestibility (Brennan & Grandison, 2012). Furthermore, denaturation of proteins also improves nutritional quality by increasing accessibility of the molecules to proteases thus making them more digestible (Brennan, 2006; Peluola‐Adeyemi et al., 2014). Feed moisture probably increases digestibility because it facilitates swelling and softening, which enhances disintegration and hydrolysis at high speed. This is supported by Njoki et al. (2014) who reported that wet cooking improves protein digestibility better than dry cooking. During heat treatment, changes in protein digestibility are influenced by the degree of formation of complexes between proteins and other grain components, and matrix disintegration which affects access of proteolytic enzymes (Muyonga et al., 2014). During extrusion, antinutritional factors which inhibit digestibility are reduced which increases digestibility. According to Muyonga et al. (2014), grain amaranth proteins have higher digestibility compared with maize and sorghum.

### 3.2 Water absorption index

Water absorption index is a measure of volume the starch occupies after swelling in excess water and is an indication of the integrity of starch (Leonel, Freitas, & Mischan, 2009; Mesquita, Leonel, & Mischan, 2013). It measures the water absorbed by starch and can be used as an index for gelatinization, an important effect of extrusion (Saini, 2015; Yang, Peng, Lui, & Lin, 2008). Quadratic Equation 9 showed that extrusion temperature (*X*_1_), speed (*X*_2_), and feed moisture content (*X*_3_) had significant effect on water absorption index of grain amaranth flour. Interaction and quadratic effects of the process variables were also observed.

(9) $$\text{Water}\;\text{absorption}\;\text{index}=1.97+0.0456X_{1}-0.2217X_{2}-0.0654X_{3}+0.0533X_{1}X_{2}+0.2783X_{1}X_{3}-0.0363X_{2}X_{3}-0.2867X_{1}^{2}+0.3790X_{2}^{2}$$

**Figure 2:**
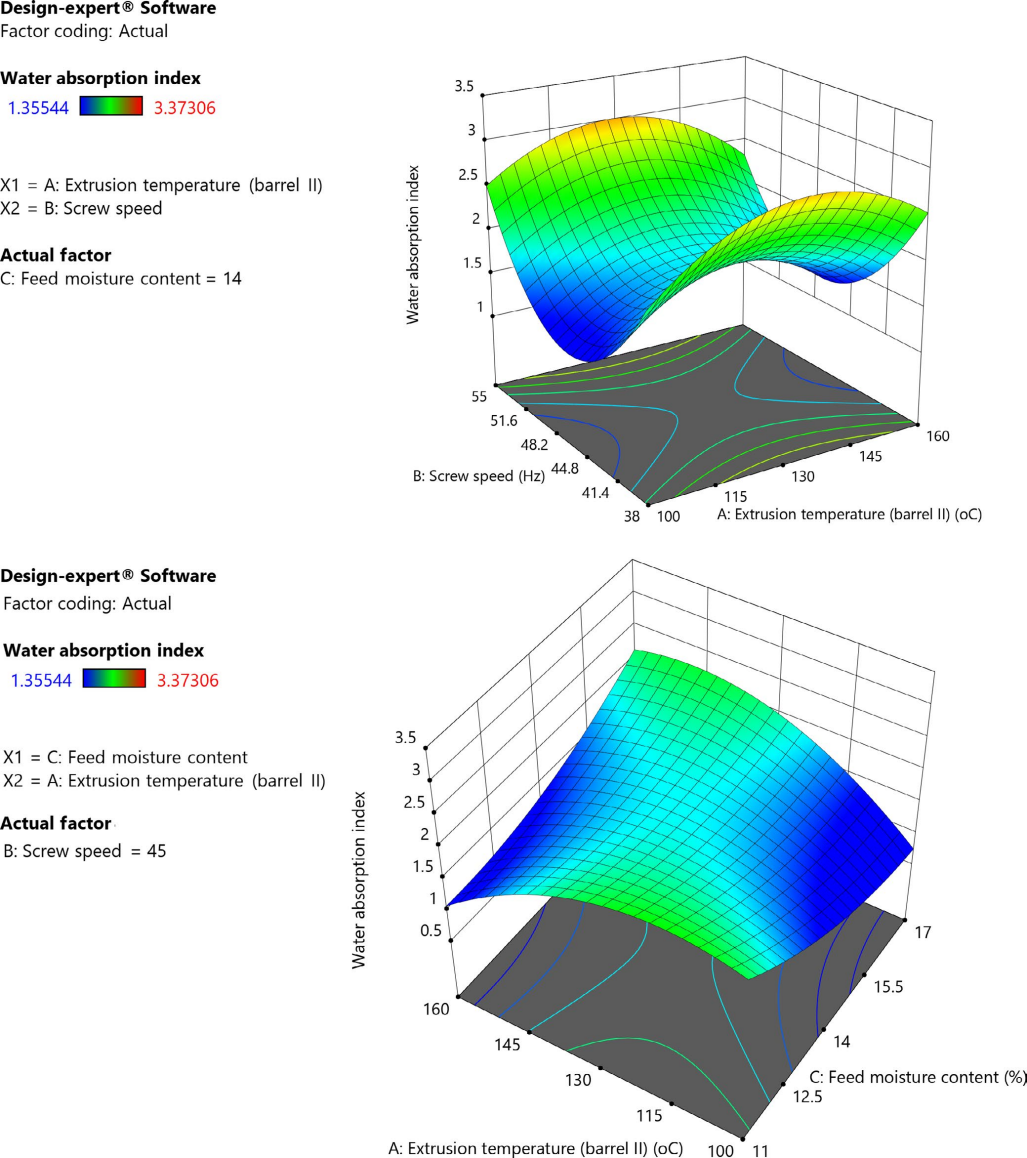
(I) Response surface plot showing effect of extrusion temperature, screw speed, and feed moisture content on water absorption index of grain amaranth flour (Constant moisture, 14%). (II) Response surface plot showing effect of extrusion temperature, screw speed, and feed moisture content on water absorption index of grain amaranth flour (Constant screw speed, 45 Hz)

The model was significant (*p *< .01) and had a nonsignificant lack of fit (*p *= .88). Extrusion temperature had a positive linear but negative quadratic effect on WAI while extrusion speed and feed moisture content both had negative linear effects. Increase in extrusion temperature resulted in increase in WAI of grain amaranth flour in the low temperature range (Figure 2). However, further increase to higher temperatures results in decreased WAI (Equation 9). According to Simons (2013) and Hernández‐Nava, Bello‐Pérez, San Martín‐Martínez, Hernández‐Sánchez, and Mora‐Escobedo (2011), extrusion typically causes gelatinization of starch, which increases water‐holding properties. The unfolding and loosening of biopolymer chains enhance availability and easier accessibility of structures by water molecules thus, the increase in WAI may be attributed to uncovering of hydrophilic groups (Marzec & Lewicki, 2006). However, further increase in extrusion temperature lowers the WAI due to formation of complexes between the soluble proteins and sugars through Maillard reactions (Mburu, Gikonyo, Kenji, & Mwasaru, 2011). On the other hand, increasing extrusion speed and feed moisture resulted in decrease of WAI of flour (Figure 2). The increase in WAI with lower feed moisture could be due to increased depolymerization of polysaccharides at low moisture content (Obradović, Babić, Šubarić, Aćkar, & Jozinović, 2014). Depolymerization of polysaccharides increases with increase in shear stress and lower moisture content of raw material (Esposito et al., 2005; Moscicki, 2011). Earlier studies have also reported lower WAI for starchy materials extruded at high moisture content (Alam, Kumar, & Khaira, 2015; Charunuch, Limsangouan, Praset, & Butsuwan, 2011). Charunuch et al. (2011) attributed the low WAI for materials extruded under high feed moisture to low extent of gelatinization due to loss of thermal energy during extrusion reduction. Water absorption index is an indication of the ability of flour to absorb and retain water (Narbutaite, Makaravicius, Juodeikiene, & Basinskiene, 2008). In complementary foods, high water absorption index is undesirable because it contributes to dietary bulk (Afam‐Anene & Ahiarakwem, 2014). During cooking, foods with high WAI absorb large amount of water to form voluminous low energy and nutrient food (Omueti et al., 2009). Therefore, the low water absorption values observed in this study are desirable as it is appropriate for making thinner gruels which have high caloric density per unit volume (Ijarotimi & Oluwalana, 2013). This is particularly crucial during processing of flours for complementary feeding.

### 3.3 Water solubility index

Water solubility index (WSI) measures soluble components from starch after extrusion and is an indication of molecular degradation (Milán‐Carrillo et al., 2012; Saini, 2015). There was a quadratic relation between extrusion conditions and water solubility index of grain amaranth, and the relation is shown by Equation 10. The model was significant (*p *= .036) and had a nonsignificant lack of fit (*p *= .81). Positive coefficients of *X*_2_ and *X*_3_ indicate that extrusion speed and feed moisture content had positive linear effects on water solubility index whereas extrusion temperature had negative linear but positive quadratic effect on grain amaranth flour WSI. Negative interaction existed between extrusion temperature and feed moisture content while positive interaction existed between extrusion speed and feed moisture.

(10) $$\text{Water}\;\text{solubility}\;\text{index}=0.5536-0.0051X_{1}+0.0186X_{2}+0.0033X_{3}+0.0019X_{1}X_{2}-0.0254X_{1}X_{3}++0.0040X_{2}X_{3}+0.0257X_{1}^{2}-0.0346X_{2}^{2}$$

**Figure 3:**
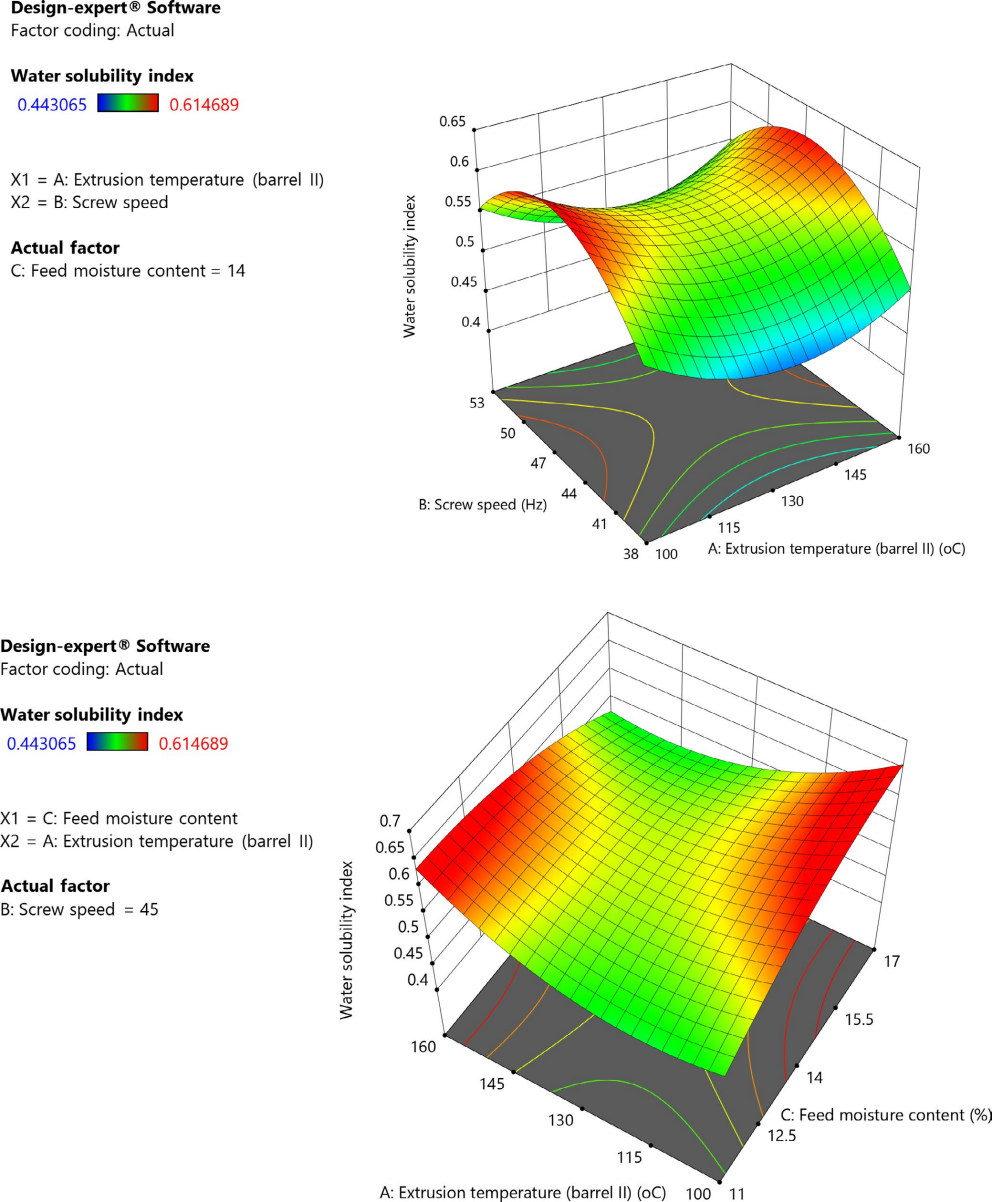
(I) Response surface plot showing effect of extrusion temperature, screw speed, and feed moisture content on water solubility index of grain amaranth flour (Constant moisture, 14%). (II) Response surface plot showing effect of extrusion temperature, screw speed, and feed moisture content on water solubility index of grain amaranth flour (Constant screw speed, 45 Hz)

Increase in extrusion temperature was associated with decrease in WSI; however, higher temperatures increased WSI of GA flour (Figure 3). This could be attributed to dextrinization of starch which could have increased with extrusion temperature and feed moisture (Peluola‐Adeyemi et al., 2014). Besides gelatinization which results into release of amylose and amylopectin, dextrinization and other reactions also occur during extrusion leading to formation of low molecular weight compounds which influence WSI (Mesquita et al., 2013). Although Gui, Gil, and Ryu (2012) reported dextrinization as dominant during the extrusion process, Chang and Ng (2011) reported that at higher temperatures, the combination of thermal and mechanical energies fully cooks the starch leading to greater degree of starch gelatinization and degradation. This could explain the quadratic effect of higher temperature on WSI. According to Yang et al. (2008), WSI indicates the degree of molecular degradation and measures extent of starch conversion during extrusion, that is, the quantity of soluble polysaccharide released from the starch. In contrast, increasing extrusion speed and feed moisture content also increased WSI. At low feed moisture content, dextrinization could have occurred easily thus increasing soluble starch and hence WSI (Gui et al., 2012). The increase in WSI with extrusion speed could be due to mechanical degradation of starch molecules resulting in increased solubility. According to Mezreb, Goullieux, Ralainirina, and Queneudec (2003), increasing screw speed induces an increase in specific mechanical energy which mechanical shear degrades macromolecules, thus decreases molecular weight of starch granules hence increasing WSI. Compaoré, Nikièma, Bassole, Savadogo, and Hounhouigan (2011) asserted that WSI increases because high molecular weight carbohydrates and proteins are hydrolyzed into simpler components. Gui et al. (2012) also reported similar effect of screw speed and feed moisture on WSI. Njoki and Faller (2001) reported that high WSI can be used to predict the ease of digestion of complementary foods by infants.

### 3.4 Bulk density

Functional properties are important because they determine the application of food materials for numerous food products (Awolu, Omoba, Olawoye, & Dairo, 2016). Bulk density (BD) is an indication of the load that the sample can carry when resting directly on one another (Ijarotimi & Oluwalana, 2013). The effect of extrusion conditions on bulk density of grain amaranth flour is represented by Equation (11).

(11) $$\text{Bulk}\;\text{density}=0.6544-0.0036X_{1}-0.0360X_{2}-0.0054X_{3}-0.0108X_{1}X_{2}+0.0050X_{1}X_{3}-0.0361X_{1}^{2}+0.0238X_{2}^{2}$$

**Figure 4:**
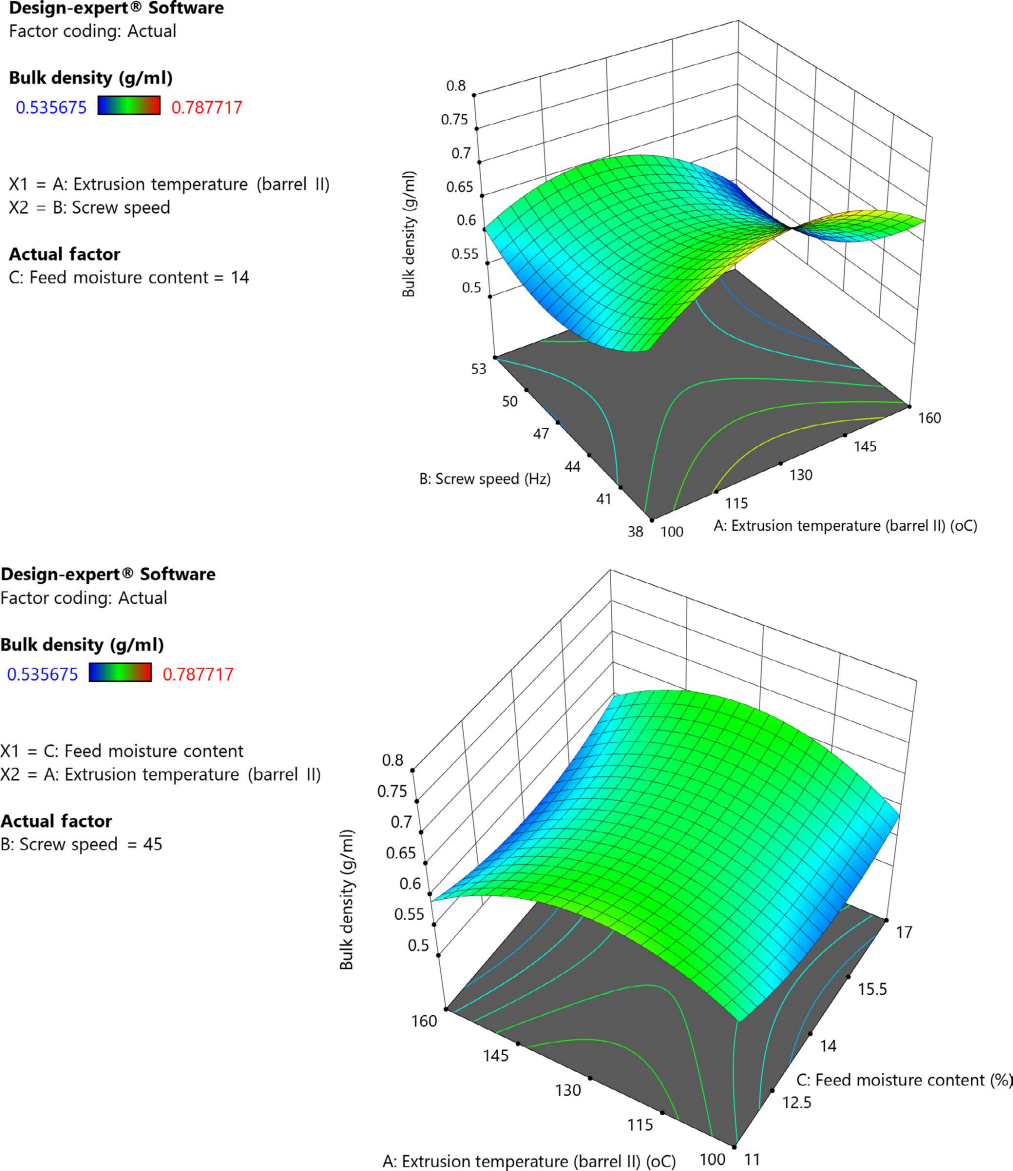
(I) Response surface plot showing effect of extrusion temperature, screw speed, and feed moisture content on bulk density of grain amaranth flour (Constant moisture, 14%). (II) Response surface plot showing effect of extrusion temperature, screw speed, and feed moisture content on bulk density of grain amaranth flour (Constant screw speed, 45 Hz)

Extrusion temperature, speed, and feed moisture had negative linear effect on bulk density of grain amaranth flour with interactions between process variables (Figure 4). The model was significant (*p *< .001) and had a nonsignificant lack of fit (*p *= .73). Temperature had a negative, while speed had a positive quadratic effect on BD (Equation 11). The negative effect of temperature on BD could be attributed to effect of heating. Increasing extrusion temperature increases the degree of superheated water in the extruder which enhances formation of bubbles and reduces melt viscosity hence leading to reduced density (Gui et al., 2012). Additionally, increase in BD with temperature could also be due to starch gelatinization. Increase in gelatinization increases volume of extruded products which results in a decrease in BD (Alam et al., 2015). This is supported by the suggestion that the structure of starch polymers influences bulk density and loose polymer structure results in low bulk density (Alawode, Idowu, Adeola, Oke, & Omoniyi, 2017). Increasing screw speed and feed moisture decreases BD because high screw speed lowers the melting viscosity and increases elasticity of the mixture which reduces BD (Ding et al., 2005). The quadratic effect of screw speed indicates that BD decreased with increase in screw speed; however, further increase in speed causes increase in BD. In a study on extrusion of a mixture of rice, lentils, and carrot pomace, Alam et al. (2015) also observed a negative relationship between bulk density and extrusion temperature. Bulk density is influenced by flour particle size and determines the packaging requirements and material handling of the flour (Alawode et al., 2017; Ijarotimi & Oluwalana, 2013). It measures flour heaviness and indicates that the volume of the flour in a package will not excessively reduce during storage (Alawode et al., 2017). During packaging, a large free space is undesirable because it creates a large reservoir for oxygen however lower bulk density result in greater oxygen transmission in the packed food (Adepeju et al., 2014; Omueti et al., 2009). Decrease in bulk density will reduce packaging and transportation costs (Bolaji, Oyewo, & Adepoju, 2014; Inyang & Effiong, 2016).

### 3.5 Viscosity

Viscosity is an important attribute which indicates increase or decrease of bulk of a cooked product and affects taste intensity (Mburu et al., 2011). Viscosity was determined as the final viscosity of the RVA, since the RVA can be used to determine the viscosity of a sample exposed to different temperatures. Final viscosity measures reassociation of starch and for extruded products, it depends on the structural modifications of granules and molecules (Hernández‐Nava et al., 2011). Quadratic Equation 12 shows positive coefficients of *X*_1_ and *X*_3_ but negative coefficients of *X*_2_, with interactions.

(12) $$\text{Viscosity}=162.60+6.86X_{1}-30.13X_{2}+0.7584X_{3}-6.31X_{1}X_{2}+19.52X_{1}X_{3}-8.57X_{2}X_{3}+39.02X_{2}^{2}$$

**Figure 5:**
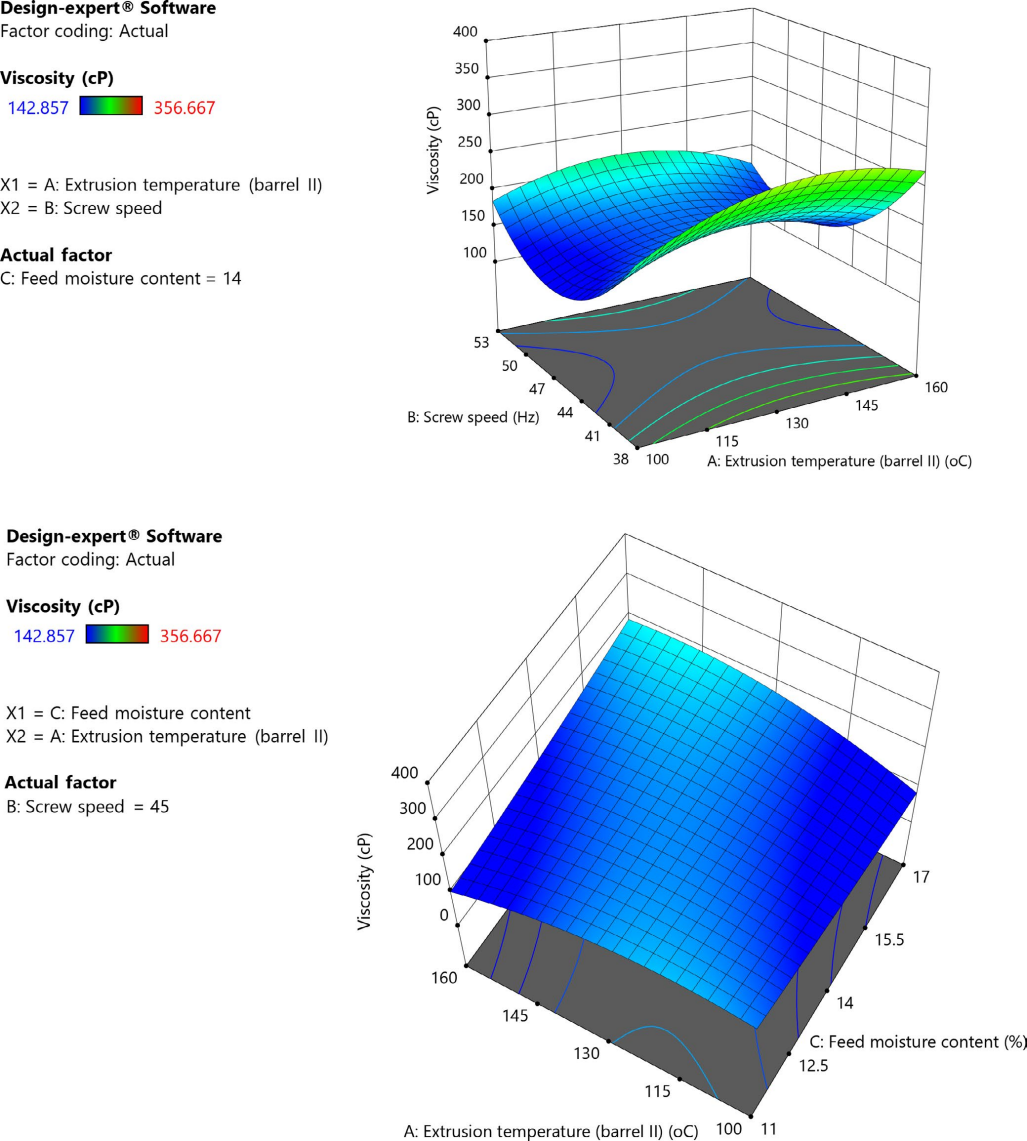
(I) Response surface plot showing effect of extrusion temperature, screw speed, and feed moisture content on viscosity of grain amaranth porridge made with mass ratio of 1:5, flour:water (Constant moisture, 14%). (II). Response surface plot showing effect of extrusion temperature, screw speed, and feed moisture content on viscosity of grain amaranth porridge (made with mass ratio of 1:5, flour:water)

Extrusion temperature and feed moisture had positive linear effect on viscosity of grain amaranth porridges. The model was significant (*p *< .001) and had a nonsignificant lack of fit (*p *= .30). However, extrusion speed had negative linear effect but positive quadratic effect on viscosity. Increase in viscosity with temperature could be due to gelatinization of starch. Increase in viscosity results from the swelling and recrystallization of native starch (Adegunwa, Adebowale, Bakare, & Kalejaiye, 2014); therefore, gelatinization which occurs during extrusion causes a decrease in native starch which hinders increase in viscosity. According to Martínez, Calviño, Rosell, and Gomez (2014), extrusion causes swelling and rupture of starch granules, destroying the organized granule structure completely or partially. Increase in viscosity with temperature could be attributed to disintegration of starch granules which increases susceptibility to hydration (Muyonga et al., 2014). Screw speed had a more significant effect on viscosity than temperature or feed moisture as evidenced by the higher coefficient of *X*_2_ and the contours on the response surface plot (Figure 5). Increasing screw speed reduces gelatinization because of decrease of residence time in the extruder which results in low swelling and volume (Obradović et al., 2014; Yu, Ramaswamy, & Boye, 2013). Leonel et al. (2009) reported low viscosity for cassava extruded with low feed moisture and attributed this to increased frictional damage especially at high screw speed. Generally, low viscosities were observed during the study which indicates that on cooking and cooling, the flours would form low viscosity gruels (Omueti et al., 2009). This implies that it is impossible to prepare gruels with relatively high solids content, and therefore high caloric density per unit volume while maintaining drinkable consistency (Ikujenlola & Fashakin, 2005; Otegbayo, Aina, Asiedu, & Bokanga, 2006). Low viscosity is required to make the food easy to consume by infants (Mburu et al., 2011).

### 3.6 Sensory acceptability

The quadratic model for sensory acceptability in terms of coded levels of the variables is shown in Equation 13.

(13) $$\text{Sensory}\;\text{acceptability}=6.42+0.0997X_{1}+0.1824X_{2}-0.1048X_{3}+0.0170X_{1}X_{2}+0.2784X_{1}X_{3}+0.0170X_{2}X_{3}+0.1469X_{1}^{2}-0.1700X_{3}^{2}$$

**Figure 6:**
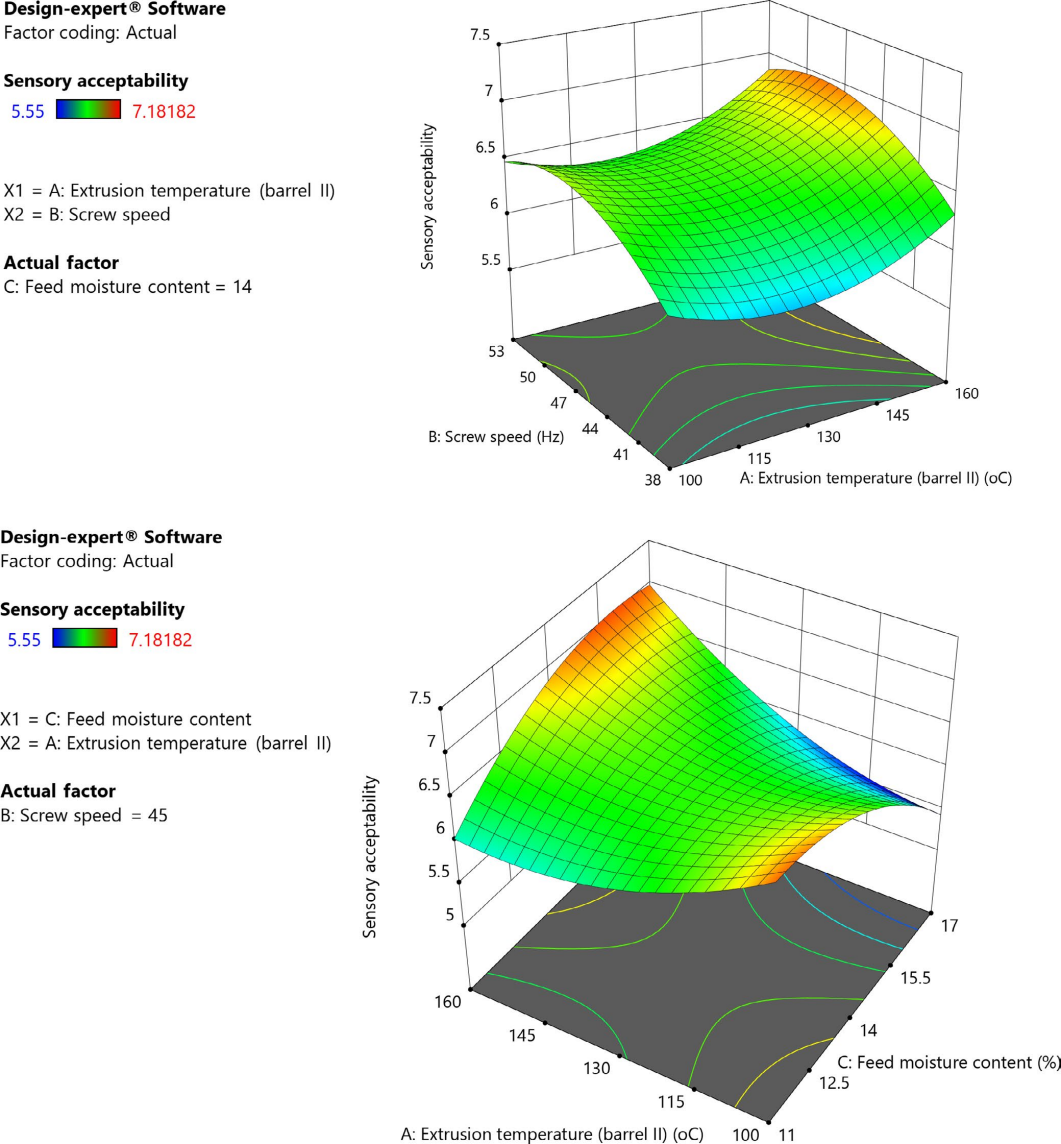
(I) Response surface plot showing effect of extrusion temperature, screw speed, and feed moisture content on response surface plot showing effect of extrusion temperature, extrusion speed, and feed moisture content on sensory acceptability of grain amaranth porridge made with flour to water mass ratio of 1:3 (Constant moisture, 14%). (II) Response surface plot showing effect of extrusion temperature, screw speed, and feed moisture content on response surface plot showing effect of extrusion temperature, extrusion speed, and feed moisture content on sensory acceptability of grain amaranth porridge made with flour to water mass ratio of 1:3 (Constant screw speed, 45 Hz)

Extrusion temperature and speed had positive linear effect on sensory acceptability while feed moisture content had negative linear and quadratic effect on sensory acceptability (Figure 6). Interactions between the process variables were observed. The model was significant (*p *= .012) with nonsignificant (*p *= .4) lack of fit, and the adequate prediction ratio showed a sufficient signal implying that the model could be used to navigate the design space. Gbenyi, Nkama, Badau, and Idakwo (2016) demonstrated the potential of using sensory scores in optimization of processes. In this study, sensory acceptability of grain amaranth porridge increased with temperature and speed. However, further increase in temperature and screw speed resulted in a decrease in acceptability. Low extrusion temperature and screw speed resulted in low sensory acceptability of grain amaranth porridge. During extrusion, low speed resulted into porridges with a browner color which panelists described as having a burnt taste. During extrusion, dextrinization and Maillard reactions occur, which causes formation of melanoidins and in turn modifies color of flours (Martínez et al., 2014). These reactions not only affect color but also the taste and flavor of the product, affecting overall product acceptability. High speed extrusion is associated with short extrusion times, and this seems to result in products with desirable sensory attributes. Lower feed moisture content increased acceptability probably due to enhanced dextrinization, which leads to formation of dextrins and other complexes that contribute to the flavor, taste, and color. However, the interaction between screw speed and the other variables indicates the importance of exposure time. Chemical reactions that occur during extrusion are important because they impart desirable sensory qualities such as flavor and color. Filli et al. (2012) reported that color changes in extruded products are due to pigment decomposition and chemical reactions such as caramelization of carbohydrates. Despite their positive effects, these reactions, if uncontrolled, negatively affect sensory attributes. The interaction effects indicate the need to consider the different processing variables together when designing extrusion protocols.

### 3.7 Optimization of extrusion conditions

Optimization of the process variables was carried out using the numerical method. Desired goals were assigned for all the parameters to obtain optimal values for the responses. During desirability determination, protein digestibility and sensory acceptability were maximized, WAI and WSI were kept in range, while viscosity and bulk density were minimized. The extrusion variables selected as optimal were those that resulted in the highest desirability (0.705) (within acceptable range for desirability). The optimal extrusion conditions were as follows: extrusion temperature of 150°C, extrusion speed of 50 Hz, and feed moisture content of 14.41%. These extrusion conditions resulted in instant grain amaranth flour with IVPD of 81.87%, WAI of 1.92, WSI of 0.55, bulk density of 0.59 (g/ml), final viscosity of 174.56 cP (14.55 RVU), and overall sensory acceptability score of 6.69.

### 3.8 Validation of results

The suitability of the developed model for prediction was tested by comparing the predicted and experimental values (Table 2). *t* Test showed that the predicted values for PD, WAI, and sensory acceptability were not statistically different from experimental values. Predicted value for BD was higher than experimental value while the predicted viscosity was significantly lower than experimental value. The observed differences between predicted and experimental values may be attributed to difference in physical variables such as humidity, room temperature, and experimental variations (Scheuer et al., 2016). The results, however, still show that response surface models correctly predicted more than half the variables, including sensory acceptability, a key attribute with respect to uptake of product. This technique is therefore still useful for optimization especially when used in combination with other techniques.

**Table 2:** Predicted and experimental values of the model

Response	Predicted value	Experimental value	*t* Test	Deviation	Relative deviation (%)	Prediction error (%)
Protein digestibility (PD)	81.87	77.85	0.052	−4.02	−5.11	−4.91
Bulk density	0.59	0.51 ± 0.01	0.016^a^	−0.08	−15.69	−13.56
Water absorption index (WAI)	1.92	1.60 ± 0.07	0.056	−0.32	−20.00	−16.67
Water solubility index (WSI)	0.55	0.67 ± 0.01	0.02^a^	0.12	17.91	21.81
Viscosity	174.56	237 ± 4.51	0.0068^a^	62.44	26.35	35.77
Sensory acceptability	6.69	7.36 ± 0.81	0.11	0.67	9.10	10.01

a Significantly different.

## 4 CONCLUSION

The study showed that extrusion parameters of temperature, screw speed, and feed moisture content together with their interactions affected the properties of extruded grain amaranth, with extrusion speed having the most significant effect. Response surface methodology was successfully used to optimize extrusion of GA flour for complementary feeding. The optimal extrusion conditions for grain amaranth flour were extruding at a temperature of 150°C (heating area II) at a speed of 50 Hz with feed moisture content of 14.41%. Porridge from flour made using optimal conditions was found to be highly acceptable and to exhibit relatively low bulk density. The above conditions can therefore be used for producing instant grain amaranth flour suitable for use in complementary foods.

## CONFLICT OF INTEREST

No conflict of interest was declared by the authors.

## ETHICAL APPROVAL

This study does not involve any human or animal testing.
